# The putative tumor activator ARHGEF3 promotes nasopharyngeal carcinoma cell pathogenesis by inhibiting cellular apoptosis

**DOI:** 10.18632/oncotarget.8283

**Published:** 2016-03-23

**Authors:** Tian-Hao Liu, Fang Zheng, Mu-Yan Cai, Lin Guo, Huan-Xin Lin, Jie-Wei Chen, Yi-Ji Liao, Hsiang-Fu Kung, Yi-Xin Zeng, Dan Xie

**Affiliations:** ^1^ Sun Yat-Sen University Cancer Center, The State Key Laboratory of Oncology in South China, Collaborative Innovation Center for Cancer Medicine, Guangzhou, China; ^2^ Department of Oncology, Sun Yat-Sen Memorial Hospital, Sun Yat-Sen University, Guangzhou, Guangdong, China; ^3^ Medical Research Center, Guangdong Provincial Key Laboratory of Malignant Tumor Epigenetics and Gene Regulation, Sun Yat-Sen Memorial Hospital, Sun Yat-Sen University, Guangzhou, China; ^4^ Department of Nasopharyngeal Cancer, Sun Yat-Sen University Cancer Center, Guangzhou, China; ^5^ Department of Pathology, Sun Yat-Sen University Cancer Center, Guangzhou, China

**Keywords:** nasopharyngeal carcinoma, ARHGEF3, BIRC8, apoptosis, pathogenesis

## Abstract

Nasopharyngeal carcinoma (NPC) is one of the most prevalent forms of highly invasive malignancy in Southern China and Southeast Asia. The pathogenesis of NPC is a multistep process driven by the acquisition of numerous genetic abnormalities. We investigated the potential oncogenic role of the Rho-guanine nucleotide exchange factor 3 gene, *ARHGEF3*, in NPC pathogenesis. Expression levels of *ARHGEF3* were frequently up-regulated in NPC cell lines and tissues. In a large cohort of clinical NPC tissues high expression of *ARHGEF3* was positively associated with an increased T status, distant metastasis, and a more advanced clinical stage (*P* < 0.05). Survival analysis revealed that *ARHGEF3* expression was a significant and independent prognosis factor for NPC patients. In NPC cell lines, knockdown of *ARHGEF3* was sufficient to inhibit cell growth, motility, and invasion *in vitro*, whereas ectopic overexpression of *ARHGEF3* substantially enhanced NPC cells tumorigenesis and metastasis *in vivo*. Depletion of *ARHGEF3* in NPC cells dramatically promoted caspase-3 induced apoptosis and an anti-apoptosis factor, *BIRC8*, was identified as a critical downstream target of the *ARHGEF3*. Our findings suggest that increased expression of *ARHGEF3* plays a critical oncogenic role in NPC pathogenesis by preventing cell apoptosis through the up-regulation of *BIRC8*, and *ARHGEF3* might be employed as a novel prognostic marker and effective therapeutic target for human NPC.

## INTRODUCTION

Nasopharyngeal carcinoma (NPC) is a unique head and neck cancer which is highly prevalent in Southern China and throughout Southeast Asia [1], with an incidence of 25–30 cases per 100,000 persons annually [2]. NPC pathogenesis is a multistep process driven by an accumulation of genetic alterations, including the loss of tumor suppressor genes and activation of oncogenes [3]. Malignant cell proliferation and apoptosis inhibition are the main factors influencing the development and progression of NPC and lead to the poor overall survival of NPC patients [4, 5]. It is essential to elucidate the molecular mechanisms underlying tumorigenesis and invasiveness in NPC in order to identify novel therapeutic targets and develop new modalities of treatment.

It has been reported that *ARHGEF3*, a Rho-guanine nucleotide exchange factor (GEF) upregulated in acute myeloid leukemia (AML), modulates AML differentiation through activation of RhoA and pathways directly controlled by small GTPase family proteins [6]. The human gene *ARHGEF3* is located at chromosome 3p13-21 and encodes a polypeptide of 526 amino acids with homology to neuroepithelial transforming gene 1 (NET1) [7–9]. *ARHGEF3* belongs to the family of Rho-GEFs which specifically activates two members of the Rho-GTPase family, RHOA and RHOB, and accelerates Rho-GTPase activity by conversion of GTP to GDP [10, 11]. Mutations in some members of the GEF family, such as DOCK2, DOCK8 and *ARHGEF6*, are associated with invasiveness and metastasis of human malignancies [12–15]. However, the potential roles and biological mechanisms of the GEF family gene *ARHGEF3* in human cancers have not been studied. To investigate if abnormalities in *ARHGEF3* are involved in NPC pathogenesis, we examined *ARHGEF3* protein levels in a series of carcinomatous and non-neoplastic human nasopharyngeal cells and tissues, assessed the clinicopathologic/prognostic significance of *ARHGEF3* expression in our NPC cohort, and investigated the mechanisms underlying the oncogenic and tumorigenic role of *ARHGEF3* in NPC.

We found that high expression of *ARHGEF3* in NPCs is important in the acquisition of an aggressive phenotype. Silencing *ARHGEF3* in NPC cells was sufficient to inhibit cell growth, migration, and invasion *in vitro*, while overexpression of *ARHGEF3* supported the tumorigenic and metastatic capacities of NPC cells *in vivo*. Further, we demonstrated that depletion of *ARHGEF3* in NPC cells promoted caspase3-induced apoptosis. We also identified the anti-apoptosis factor *BIRC8* as a critical downstream target of *ARHGEF3*. Collectively, our results provide an explanation for the malignant nature of NPC involving *ARHGEF3* overexpression and the underlying mechanism that links *ARHGEF3* to *BIRC8* in NPC cell apoptosis.

## RESULTS

### Analysis of *ARHGEF3* protein levels in NPC cells and nasopharyngeal tissues

**Figure 1:**
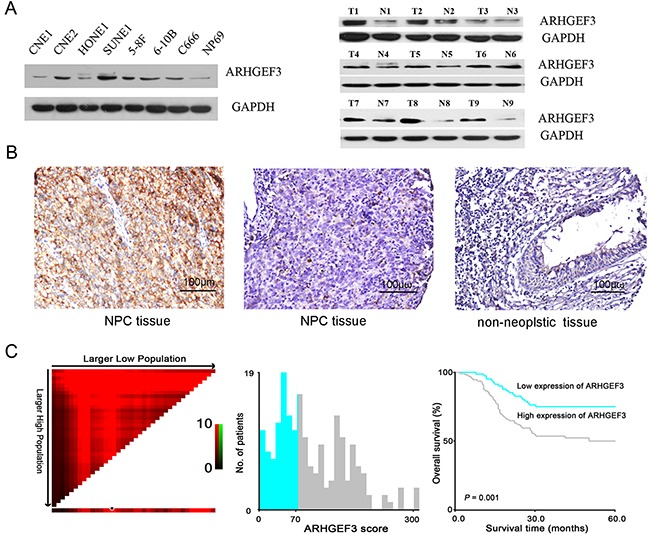
Expression of *ARHGEF3* in nasopharyngeal cell lines and tissues and its prognostic significance in nasopharyngeal carcinoma (NPC) patients **A.** Western blot showing relative levels of *ARHGEF3* protein in 8 nasopharyngeal cell lines (left). *ARHGEF3* expression was up-regulated in primary NPC tissues compared with paired non-neoplastic nasopharyngeal mucosa tissues (right). **B.** Representative immunohistochemistry images showing high expression of ARHGEF3 in one NPC tissue (case 27, left), low expression of *ARHGEF3* in another NPC tissue (case 99, middle), and negative expression of *ARHGEF3* in a non-neoplastic nasopharyngeal tissue (case 33, right). **C.** X-tile plots of the prognostic marker *ARHGEF3*. X-tile analysis was carried out on patient data from the NPC cohort. The plot shows the χ^2^ log-rank values. Panels depict the cut-off point for high expression (highlighted by the black/white circle; left), a histogram of the entire cohort (middle), and a Kaplan–Meier survival curve (right).

We analyzed endogenous *ARHGEF3* protein levels in 8 human nasopharyngeal cell lines by Western blotting and found that *ARHGEF3* was overexpressed in 5 NPC cell lines (CNE2, SUNE1, 5-8F, 6-10B and C666), while the other 2 NPC lines (CNE1 and HONE1) and the immortalized normal nasopharyngeal cell line NP69 exhibited low *ARHGEF3* protein levels (Figure 1A, left). At the same time, we found that *ARHGEF3* protein expression was higher in 8 primary NPC tissues, compared with adjacent non-neoplastic nasopharyngeal tissues. But there were no difference between the tumor and adjacent tissues in 1 case. (Figure 1A, right).

### IHC staining of *ARHGEF3* expression in NPC tissues and its correlation with NPC patients’ pathological features and survival

Using IHC staining, we observed high expression of *ARHGEF3* (Figure 1B, left) in 111 of 192 (57.8%) primary NPC tissues (Table 1). 17 cases of NPC were not informative due to unrepresentative samples or lost samples. We used the whole NPC tissue slides of these cases to improve the limitation of TMA technology in our study. Correlation analysis demonstrated that high expression of *ARHGEF3* was positively associated with an increased T status, distant metastasis, and/or a more advanced clinical stage of NPCs (*P*< 0.05, Table 1). Kaplan-Meier survival curves showed that the mean disease-free survival time in NPC patients with high expression of *ARHGEF3* was significantly shorter than in patients with low expression of *ARHGEF3* (*P*=0.001, long-rank test, Figure 1C, Table 2). Multivariate Cox proportional hazards regression analysis demonstrated that high expression of *ARHGEF3* was a significant and independent prognostic factor for poor survival of NPC patients (relative risk: 1.709, confidence interval: 1.002-2.913, *P*=0.049, Table 2).

**Table 1:** Correlation between the clinicopathological features and expression of ARHGEF3 in NPCs

	cases	ARHGEF3 protein		
		Low expression	High expression	*P* value^*^
Sex				0.532
Female	57	26 (45.6%)	31 (54.4%)	
Male	135	55 (40.7%)	80 (59.3%)	
Age at diagnosis (years)				0.299
≤ 47^†^	103	47 (45.6%)	56 (54.4%)	
> 47	89	34 (38.2%)	55 (61.8%)	
Histological classification (WHO)				0.303
Type II	50	18 (36.0%)	32 (64.0%)	
Type III	142	63 (44.4%)	79 (55.6%)	
T classification				0.044
1	23	10 (43.5%)	13 (56.5%)	
2	65	31 (47.7%)	34 (52.3%)	
3	67	32 (47.8%)	35 (52.2%)	
4	37	8 (21.6%)	29 (78.4%)	
N classification				0.203
0	38	15 (39.5%)	23 (60.5%)	
1	89	43 (48.3%)	46 (51.7%)	
2	50	20 (40.0%)	30 (60.0%)	
3	15	3 (20.0%)	12 (80.0%)	
Distant metastasis				0.005
0	152	72 (47.4%)	80 (52.6%)	
1	40	9 (22.5%)	31 (77.5%)	
Clinical stage				0.002
I	9	3 (33.3%)	6 (66.7%)	
II	50	26 (52.0%)	24 (48.0%)	
III	83	42 (50.6%)	41 (49.4%)	
IV	50	10 (20.0%)	40 (80.0%)	

* Chi-square test;

† median age.

Abbreviation: ARHGEF3, Rho guanine nucleotide exchange factor3; NPC, nasopharyngeal carcinoma; T, tumor; N, node.

**Table 2:** Univariate and multivariate analysis of different prognostic parameters in 192 patients with NPC

Variable	Univariate analysis			Multivariate analysis	
	All cases	HR (95% CI)	*P* value	HR (95% CI)	*P* value
Sex			0.868		
Female	57	1.0			
Male	135	0.959 (0.581-1.581)			
Age at surgery (years)			0.511		
≤ 47^*^	103	1.0			
> 47	89	0.856 (0.539-1.360)			
Histological classification (WHO)			0.126		
Type II	50	1.0			
Type III	142	1.577 (0.881-2.824)			
T classification			<0.0001		0.744
T1-T2	88	1.0		1.0	
T3-T4	104	2.465 (1.484-4.094)		1.106 (0.605-2.020)	
N classification			<0.0001		0.050
N0-N1	127	1.0		1.0	
N2-N3	65	2.829 (1.781-4.492)		1.668 (1.001-2.779)	
Distant metastasis			<0.0001		< 0.0001
0	152	1.0		1.0	
1	40	4.462 (2.791-7.135)		2.602 (1.575-4.298)	
Clinical stage			<0.0001		0.034
I-II	59	1.0		1.0	
III-IV	133	6.291 (2.727-14.515)		3.103 (1.087-8.859)	
ARHGEF3 expression			0.001		0.049
Low	81	1.0		1.0	
high	111	2.334 (1.395-3.905)		1.709 (1.002-2.913)	

* median age; HR indicates hazards ratio; CI indicates confidence interval.

Abbreviation: NPC, nasopharyngeal carcinoma; ARHGEF3, Rho guanine nucleotide exchange factor3; T, tumor; N, node.

### Knockdown of *ARHGEF3* suppresses NPC cell growth, migration, and invasion *in vitro*

**Figure 2:**
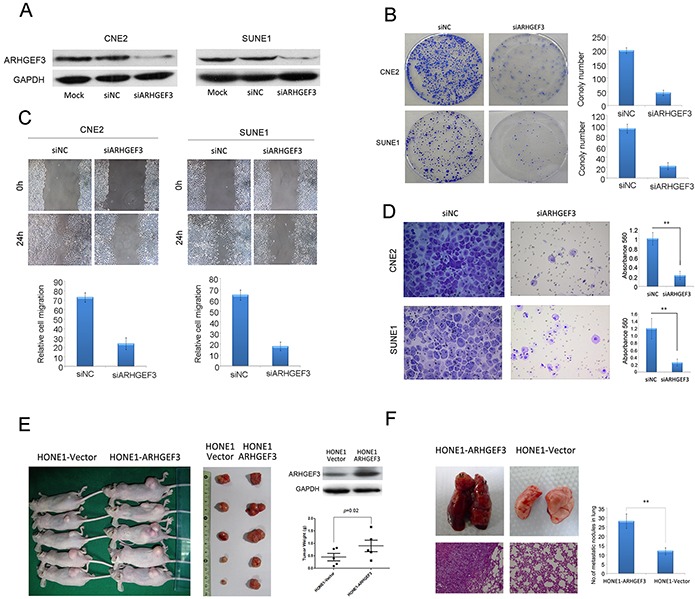
Effect of *ARHGEF3* on NPC cells colony formation, cell motility, and invasion *in vitro* and tumorigenesis and metastasis *in vivo* **A.** Western blot confirming reveals that *ARHGEF3* was efficiently knocked down by the treatment of a specific siRNA in CNE2 and SUNE1 NPC cells. **B.** Representative images of decreased colony formation in monolayer culture induced by knockdown of *ARHGEF3* in NPC cells. **C.** The wound-healing assay shows that knockdown of *ARHGEF3* substantially inhibited the migration of CNE2 and SUNE1 cells. **D.** Transwell invasion assays show that *ARHGEF3*-silenced CNE2 and SUNE1 cells had lower invasive capacity compared to control cells. Data are the mean ± SD of at least 3 independent experiments. **P< 0.01 by Student's *t* test. **E.** Western blots of *ARHGEF3* protein levels in HONE1-*ARHGEF3* and HONE1-vector cells (right, upper). Images of the xenograft tumors formed in nude mice injected with HONE1-*ARHGEF3* or HONE1-vectorcells (left and right, lower). Weights of xenograft tumors are given as mean ± SD. **, *P*=0.02 by Student's *t* test. **F.** Representative image of lungs showing metastatic nodules originating from HONE1-*ARHGEF3* or control HONE1-vector cells injected into BALB/C-nu athymic nude mice. H&E staining of lung metastatic tumors are shown (left). Quantification of metastatic nodules formed in the lungs ofmice 8 weeks after tail vein injection of HONE1-*ARHGEF3* or HONE1-vector cells (n = 5 mice per group; *P*<0.001, independent Student's *t* test, right).

The above observations prompted us to explore the biological function of *ARHGEF3* in NPC tumorigenesis and progression. The capacity for colony formation was evaluated in two NPC cell lines (CNE2 and SUNE1) that were transfected with si*ARHGEF3* or control siNC. The efficiency of *ARHGEF3* knockdown by si*ARHGEF3* was examined by Western blotting (Figure 2A). Both *ARHGEF3*-silenced CNE2 and SUNE1 cells had fewer and smaller colonies than that siNC-transfected cells (Figure 2B), indicating that depletion of *ARHGEF3* inhibits growth in NPC cells. Next, the effect of *ARHGEF3* levels on NPC cell migration and invasion capacities were characterized by the wound-healing and Matrigel invasion assays, respectively. Knockdown of *ARHGEF3* in both CNE2 and SUNE1 cells caused a dramatic suppression of cell migration and invasion abilities as compared to control cells (Figure 2C and 2D).

### Upregulated expression of *ARHGEF3* supports the tumorigenic and metastatic capacities of NPC cells *in vivo*

To investigate whether levels of *ARHGEF3* influence the tumorigenic function of NPC cells *in vivo,* we first constructed a HONE1-*ARHGEF3* cell line which stably overexpressed *ARHGEF3* (Figure 2E, right, upper) . Next, HONE1-*ARHGEF3* cells were transplanted into the backs of BALB/C-nu athymic nude mice, while HONE1-vector cells were used as a negative control (n = 5 mice per group). Thirty days after cell injection mice were sacrificed and the size and the weight of the subcutaneous tumors were examined. Tumors developed from HONE1-*ARHGEF3* cells were significantly larger and heavier (*P*=0.02) than those arising from control cells (Figure 2E). To investigate if increased expression of *ARHGEF3* in NPC cells is causative in an *in vivo* experimental metastasis model, we injected HONE1-*ARHGEF3* or control HONE1-vector cells into the tail vein of BALB/C-nu athymic nude mice (n = 5 mice per group). Eight weeks after injection, mice were killed and metastatic tumor nodules formed in lung and liver were examined. We did not detect tumor nodule formation in the livers of all mice examined, but overexpression of *ARHGEF3* significantly increased metastasis in lung (*P*<0.01, Figure 2F).

### Expression level of *ARHGEF3* influences the apoptosis of NPC cells *in vitro*

**Figure 3:**
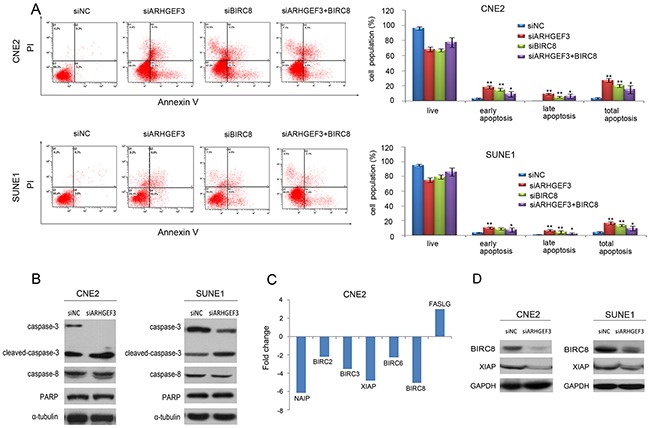
Knockdown of *ARHGEF3* promotes cellular apoptosis and regulates apoptosis-associated gene expression in NPC cells **A.** A representative image showing that knockdown of *ARHGEF3* and *BIRC8* in CNE2 and SUNE1 cells significantly increases cell apoptosis compared to control cells (A). At first, CNE2 and SUNE1 cells were transfected with si*ARHGEF3* or si*BIRC8* alone. Then in rescue experiment, three days after the transfection of si*ARHGEF3*, cells were transfected with pcDNA3.1(+)-*BIRC8*. Finally, the apoptotic assay was performed with flow cytometry. The knockdown of *ARHGEF3* and *BIRC8* in CNE2 and SUNE1 cells significantly increases cell apoptosis *compared* to control cells. Ectopic expression of *BIRC8* in CNE2 and SUNE1 cells with the knockdown of *ARHGEF3* reversed the pro-apoptotic function of si*ARHGEF3* (left). Data represent the mean ± SD of at least 3 independent experiments. **P<0.01, ***P<0.001 by Student's *t* test (right). **B.** Western blot showing that si*ARHGEF3* in CNE2 and SUNE1 cells increased levels of the active cleaved form of caspase-3 compared with siNC treatment. **C.** A total of six down-regulated genes (*BIRC2, BIRC3, BIRC6, BIRC8, NAIP* and *XIAP*) and one up-regulated gene (*FASLG*) showed more than a two-fold mRNA differential expression in si*ARHGEF3*-CNE2 cells. **D.** Knockdown of *ARHGEF3* by si*ARHGEF3* down-regulated protein levels of *BIRC8* and *XIAP* in both CNE2 and SUNE1 cells.

To further study the effect of *ARHGEF3* on NPC cell apoptosis, we transfected CNE2 and SUNE1 cells with either si*ARHGEF3* or si*BIRC8* for 48h and found that knockdown of *ARHGEF3* and *BIRC8* increased apoptosis in both CNE2 and SUNE1 cells compared with control cells. Then in rescue experiment, three days after the transfection of si*ARHGEF3*, cells were transfected with pcDNA3.1(+)-*BIRC8*. Finally, the apoptotic assay was performed with flow cytometry. Ectopic expression of *BIRC8* in CNE2 and SUNE1 cells with the knockdown of *ARHGEF3* reversed the pro-apoptotic function of si*ARHGEF3* (Figure 3A). We also determined that the levels of active, cleaved caspase-3 were substantially increased in si*ARHGEF3*-CNE2 and si*ARHGEF3*-SUNE1 cells when compared to matched control cells (Figure 3B). These data suggest that the attenuation of *ARHGEF3* expression promotes NPC cell apoptosis.

### *ARHGEF3* regulates apoptosis-related gene expressions in NPC cells

In an effort to determine the potential downstream targets of *ARHGEF3* that are involved in the promotion of NPC cell apoptosis, we compared mRNA expression profiles of si*ARHGEF3*-CNE2 cells with those of control siNC-CNE2 cells using a Human Tumor Apoptosis RT^2^ Profiler™ PCR Array containing 84 apoptosis-related genes. We identified a total of 6 downregulated genes (*NAI*P, *BIRC2*, *BIRC3, XIAP*, *BIRC6*, and *BIRC8*) and 1 upregulated gene (*FASLG*) in si*ARHGEF3*-transfected CNE2 cells, which showed more than a twofold change in mRNA levels compared to control siNC-CNE2 cells (Figure 3C and Table 3). Downregulation of *XIAP* and *BIRC8 (ILP-2)* was further validated by Western blotting assay in CNE2 and SUNE1 cells after *ARHGEF3* knockdown (Figure 3D). Further, we found a significant positive correlation between the expression of *ARHGEF3* and *BIRC8* in our large cohort of NPC tissues (*P*=0.015, Table 4). There was no significant difference in *XIAP* expression between the *ARHGEF3* high-expressing and low-expressing groups (*P* = 0.321, Table 4).

**Table 3:** List of genes differentially expressed in NPC CNE2 cells after ARHGEF3 knockdown using a human tumor apoptosis real-time PCR array

Gene	Fold Change	Location	Function
**Downregulated genes**			
ABL1	−1.47	9q34.12	Induces cell division, adhesion
AKT1	−1.37	14q32.33	Inhibits cell apoptosis
BAD	−1.40	11q13.1	Induces cell apoptosis
BAG1	−1.68	9p13.3	Inhibits cell apoptosis
BAG3	−1.83	10q26.11	Inhibits cell apoptosis
BAG4	−1.45	8p11.23	Inhibits cell apoptosis
BAK1	−1.73	6p21.31	Induces cell apoptosis
BAX	−1.65	19q13.33	Induces cell apoptosis
BCL2A1	−1.93	15q25.1	Inhibits cell apoptosis
BCL2L1	−1.63	20q11.21	Inhibits cell apoptosis
BCL2L10	−1.03	15q21.2	Inhibits cell apoptosis
BCL2L11	−1.76	2q13	Induces cell apoptosis
BCL2L2	−1.91	14q11.2	Inhibits cell apoptosis
BFAR	−1.47	16p13.11	Inhibits cell apoptosis
BID	−1.01	22q11.21	Induces cell apoptosis
BIK	−1.95	22q13.2	Induces cell apoptosis
**BIRC2**	**-2.22**	**11q22.2**	**Inhibits cell apoptosis**
**BIRC3**	**-3.55**	**11q22.2**	**Inhibits cell apoptosis**
**BIRC6**	**-2.27**	**2p22.3**	**Inhibits cell apoptosis**
**BIRC8**	**-5.04**	**6q21**	**Inhibits cell apoptosis**
BNIP1	−1.03	5q35.1	Inhibits cell apoptosis
BNIP2	−1.69	15q22.2	Inhibits cell apoptosis
BNIP3	−1.65	10q26.3	Inhibits cell apoptosis
BNIP3L	−1.60	8p21.2	Inhibits cell apoptosis
BRAF	−1.97	7q34	Inhibits cell apoptosis
CARD6	−1.07	5p13.1	Induces cell apoptosis
CARD8	−1.67	19q13.33	Induces cell apoptosis
CASP3	−1.11	4q35.1	Induces cell apoptosis
CASP4	−1.13	11q22.3	Induces cell apoptosis
CASP5	−1.58	11q22.3	Induces cell apoptosis
CASP6	−1.52	4q25	Induces cell apoptosis
CASP7	−1.56	10q25.3	Induces cell apoptosis
CASP8	−1.29	2q33.1	Induces cell apoptosis
CD40	−1.00	20q13.12	Induces cell apoptosis
CFLAR	−1.54	2q33.1	Inhibits cell apoptosis
CIDEA	−1.13	18p11.21	Induces cell programmed death
CIDEB	−1.47	14q12	Induces cell programmed death
CRADD	−1.99	12q22	Induces cell apoptosis
DAPK1	−1.02	9q21.33	Induces cell programmed death
FADD	−1.66	11q13.3	Induces cell programmed death
FAS	−1.50	10q23.31	Induces cell programmed death
GADD45A	−1.17	1p31.3	Induces cell apoptosis
HRK	−1.01	12q24.22	Induces cell apoptosis
IGF1R	−1.74	15q26.3	Inhibits cell apoptosis
LTA	−1.47	6p21.33	Induces cell apoptosis
MCL1	−1.86	1q21.3	Inhibits cell apoptosis
**NAIP**	**-6.12**	**5q13.2**	**Inhibits cell apoptosis**
NOL3	−1.45	16q22.1	Inhibits cell apoptosis
RIPK2	−1.58	8q21.3	Induces cell apoptosis
CD70	−1.88	19p13.3	T cell activator
TNFSF8	−1.26	9q32	Induces cell apoptosis
TP53	−1.89	17p13.1	Induces cell apoptosis
TP53BP2	−1.13	1q41	Induces cell apoptosis
TP73	−1.27	1p36.32	Induces cell apoptosis
TRADD	−1.38	16q22.1	Induces cell apoptosis
TRAF2	−1.27	9q34	Induces cell apoptosis
TRAF3	−1.85	14q32.32	Induces cell apoptosis
**XIAP**	**-4.8**	**Xq25**	**Inhibits cell apoptosis**
B2M	−1.23	15q21.1	Immune response
HPRT1	−1.17	Xq26.1	Nucleotide metabolism
ACTB	−1.09	7p22.1	Cytoskeleton actin
**Upregulated genes**			
APAF1	1.16	12q23.1	Induces cell apoptosis
BCL10	1.79	1p22.3	Induces cell apoptosis
BCL2	1.17	18q21.33	Induces cell apoptosis
BCLAF1	1.37	6q23.3	Induces cell apoptosis
NOD1	1.75	7p14.3	Induces cell apoptosis
CASP1	1.18	11q22.3	Induces cell apoptosis
CASP10	1.96	2q33.1	Induces cell apoptosis
CASP14	1.08	19p13.12	Induces cell apoptosis
CASP2	1.08	7q34	Induces cell apoptosis
CASP9	1.67	1p36.21	Induces cell apoptosis
CD40LG	1.44	Xq26.3	Inhibits cell apoptosis
DFFA	1.55	1p36.22	Induces DNA damage
**FASLG**	**2.99**	**1q24.3**	**Induces cell apoptosis**
LTBR	1.77	12p13.31	Inhibits cell apoptosis
PYCARD	1.72	16p11.2	Induces cell apoptosis
TNF	1.74	6p21.33	Induces cell apoptosis
TNFRSF10A	1.11	8p21.3	Induces cell apoptosis
TNFRSF10B	1.08	8p21.3	Induces cell apoptosis
TNFRSF11B	1.66	8q24.12	Induces cell apoptosis
TNFRSF1A	1.06	12p13.31	Induces cell apoptosis
TNFRSF21	1.40	6p21.1	Induces cell apoptosis
TNFRSF25	1.09	1p36.31	Induces cell apoptosis
CD27	1.39	12p13.31	Induces cell apoptosis
TNFRSF9	1.51	1p36.23	Induces cell apoptosis
TNFSF10	1.59	3q26.31	Induces cell apoptosis
RPL13A	1.14	19q13.3	Protein metabolism
GAPDH	1.02	12p13.31	Glycometabolism

Abbreviation: NPC, nasopharyngeal carcinoma; ARHGEF3, Rho guanine nucleotide exchange factor3.

**Table 4:** Correlation between expression of ARHGEF3 and that of BIRC8 and XIAP in 192 patients with NPC

	All cases	BIRC8 expression			XIAP expression		
		Low	High	*P* value^*^	Low	High	*P* value^*^
ARHGEF3 expression				0.015			0.321
Low	81	48 (59.3%)	33 (40.7%)		46 (56.8%)	35 (43.2%)	
High	111	46 (41.4%)	65 (58.6%)		55 (49.5%)	56 (50.5%)	

* Chi-square test.

Abbreviation: NPC, nasopharyngeal carcinoma; ARHGEF3, Rho guanine nucleotide exchange factor3.

## DISCUSSION

*ARHGEF3* is a key activator of Rho-GTPases including, in particular RhoA, Rac1, and CDC42, which function as molecular switches in a variety of cellular signaling pathways [16, 17]. Increasing evidence suggests that Rho-GTPases are frequently deregulated during tumor progression, which promotes malignant phenotypes in cancer cells and is correlated with poor patient prognosis [18–20]. However, the molecular status of *ARHGEF3* and its potential function in the underlying mechanisms of NPC are unclear.

In the present study, we demonstrated that the majority of human NPC cell lines and tissues expressed high levels of endogenous *ARHGEF3* protein compared to control non-neoplastic nasopharyngeal cells and tissues. These findings suggest that upregulated expression of *ARHGEF3* provides a selective advantage in NPC pathogenic processes.

Our analyses also found that high *ARHGEF3* expression in our NPC cohorts was positively correlated with tumor T status, distant metastasis, and advanced clinical stage, suggesting that high expression of *ARHGEF3* facilitates a malignant phenotype in NPC. Further, we observed that expression of *ARHGEF3* was a strong and independent prognostic predictor for NPC patients. These findings underscore a potentially important role of *ARHGEF3* in the development and progression of NPC, and suggest that examination of *ARHGEF3* expression by IHC could be used as an additional tool in identifying those NPC patients at increased risk of tumor growth and/or metastasis.

Our investigation of the mechanisms through which *ARHGEF3* regulates NPC cell malignancy demonstrated that knockdown of *ARHGEF3* in CNE2 and SUNE1 NPC cells dramatically repressed cell growth, migration, and invasion *in vitro*. In contrast, *ARHGEF3* overexpression in HONE1 NPC cells induced cell tumorigenicity *in vivo*. Moreover, in a tail vein injection mouse model of cancer metastasis ectopic overexpression of *ARHGEF3* in HONE1 cells led to a significant increase in the number of metastatic lung lesions. These data support our emerging view that increased *ARHGEF3* expression is a critical molecular event in the process of NPC pathogenesis.

It has been reported that cancer cells exhibit deficiencies in the induction of apoptosis, resulting in accelerated tumor development and reduced responsiveness to anti-cancer therapies [21–23]. Conversely, increased apoptosis in cancer cells may provide therapeutic benefits, especially in apoptosis-defective cancers [24, 25]. In the current study, we found that silencing *ARHGEF3* in NPC cells could induce apoptosis, as measured by an increased percentage of annexin V positive cells and increased cleavage of caspases3. Gene expression profiling also revealed that silencing of *ARHGEF3* resulted in downregulation of a number of genes, most notably *BIRC8*. Further, we did observe a significant positive correlation between expression of *ARHGEF3* and *BIRC8* in our large cohort of NPC clinical tissues.*BIRC8 (ILP-2)* belongs to the inhibitors of apoptosis protein (IAP) family, which are apoptosis inhibitors that may protect against apoptotic stimuli and suppress apoptosis [26]. It has been reported that *BIRC8* exerts its effects by association with an inhibition of specific caspases [27]. In addition, studies have documented that *BIRC8 (ILP-2)* is a tumor biomarker and promotes cancer progression [28, 29]. These results suggest that *ARHGEF3* regulates cell apoptosis via control of BIRC8 expression, which is in turn involved in attenuation of caspases3-induced apoptosis in the pathogenesis of NPC.

Our study demonstrates, for the first time, the protein expression dynamics of *ARHGEF3* in a large cohort of clinical NPC tissues. High expression of *ARHGEF3* may be important in tumorigenesis and acquisition of a poor prognostic phenotype of human NPC. Importantly, our functional and mechanistic studies suggest an important oncogenic role for *ARHGEF3* in the suppression of NPC cell apoptosis by regulating *BIRC8* expression and caspases3-induced apoptosis, activities that might be responsible for the development and progression of human NPCs.

## MATERIALS AND METHODS

### Nasopharyngeal cell lines and tissue specimens

Seven human NPC cell lines (CNE1, CNE2, HONE1, SUNE1, 5-8F, 6-10B and C666) and one immortalized normal nasopharyngeal cell line (NP69) were cultured in RPMI-1640 supplemented with 10% fetal bovine serum. 192 specimens of NPC and 50 specimens of non-neoplastic nasopharyngeal mucosa were collected at Sun Yat-Sen University Cancer Center, Guangzhou, China, between January 1991 and August 2000. A nasopharyngeal tissue microarray (TMA) was then constructed. In addition, 9 pairs of fresh NPC tissues and adjacent non-neoplastic nasopharyngeal mucosa specimens were collected at Guangdong Provincial People's Hospital (Guangzhou, China) in 2012. None of the NPC patients had received preoperative radiation or chemotherapy before diagnosis. A pathological diagnosis for all specimens was confirmed according to the 2005 WHO histological classification of NPC. Tumor stage was defined according to the criteria of the sixth edition of the TNM classification of the Union for International Cancer Control (UICC, 2002). The study was approved by the Institute Research Medical Ethics Committee of Sun Yan-Sun University Cancer Center (Guangzhou, China).

### Western blotting assay

Equal amounts of whole cell and tissue lysates were resolved by SDS-polyacrylamide gel electrophoresis and electrotransferred on a polyvinylidenedifluoride membrane (Pall Corp., Port Washington, NY). The samples were then incubated with primary antibodies against *ARHGEF3* (Abgent Laboratories, San Diego, CA), caspase-3, cleaved caspase-3, caspase-8 (BD Biosciences, San Jose, CA), *PARP, NAIP*(Abcam, Cambridge, UK), *BIRC2, BIRC3, XIAP, BIRC6, BIRC8* (Proteintech, Chicago, IL), *FASLG* (Abnova, Taibei City, Taiwan), α-tubulin (Santa Cruz Biotech, Dallas, TX), or *GAPDH* (BD Biosciences, San Jose, CA). The immunoreactive signals were detected with an enhanced chemiluminescence reagent kit (Amersham Biosciences, Uppsala, Sweden). The procedures were conducted in accordance with the manufacturer's instructions.

### Immunohistochemistry (IHC) staining

IHC studies were performed using a standard streptavidin biotin-peroxidase complex method. For antigen retrieval, TMA slides were microwave treated in 10 mM citrate buffer (pH 6.0) for 10 min. The TMA slides were incubated with anti-*ARHGEF3* (1:100 dilution; Abgent Laboratories, San Diego, CA), in a moist chamber overnight at 4°C. A negative control was obtained by replacing the primary antibody with a normal rabbit IgG.

A semi-quantitative scoring criterion for IHC of *ARHGEF3* was used, in which both staining intensity and positive areas were recorded. A staining index (values 0 to 12) obtained as the intensity of positive staining (negative = 0, weak = 1, moderate = 2, or strong = 3 scores) and the proportion of immunopositive cells of interest (< 25% = 1, 25 to 50% = 2, 51% to 75% = 3, > 75% = 4 scores) were calculated. Because the staining index of expression of *ARHGEF3* in all 50 non-neoplastic nasopharyngeal mucosa tissues was no more than 4, we designated staining index scores of 0-4 as “low” expression of *ARHGEF3* (Figure 1B right) and staining index scores of 6-12 was as “high” expression of *ARHGEF3* (Figure 1B left). The cutoff scores for determining “high” level expression of *XIAP* and *BIRC8 (ILP-2)* were determined at staining index scores of >4 and >6, respectively.

### Small interfering RNA (siRNA)

CNE2 and SUNE1 cells were cultured in six-well plates. The cells were transfected with anti-*ARHGEF3* siRNA or anti-control siRNA (Ambion, Austin, Texas) using Lipofectamine 2000 reagent (Invitrogen, Carlsbad, CA) according to the manufacturer's instructions. The gene silencing effect was measured by Western blotting 48 h post-transfection.

### Colony formation assay

Five hundred infected cells were placed in a fresh six-well plate and maintained in RPMI1640 containing 10%FBS for 10 days. Colonies were fixed with methanol and stained with 0.1% Giemsa in 20% methanol for 15 minutes.

### Wound-healing and matrigel invasion assays

Cell migration was assessed by measuring the movement of cells into a scraped cellular area created by 200μL pipette tip. The spread of wound closure was observed after 48h and photographed under a microscope. We measured the fraction of cell coverage across the line to calculate migration rate.

For invasion assays, 1×10^4^ cells were added to a Matrigel invasion chamber (BD Biosciences, Becton Dickson Labware, Franklin Lakes, NJ) present in the insert of a 24-well culture plate. FBS was added to the lower chamber as a chemoattractant. After 48h, the non-invading cells were gently removed with a moist cotton swab. Invasive cells located on the lower side of the chamber were fixed with paraformaldehyde and then stained with crystal violet, air dried, and photographed. For colorimetric assays, the samples were treated with 150μL 10% acetic acid and absorbance was measured with a spectrophotometer at 560nm.

### Plasmid constructs and transfection

*ARHGEF3* and *BIRC8* complementary DNA (Fulengen, Guangzhou, China) was cloned into a pcDNA3.1 plasmid. HONE1 Cells were transfected with pcDNA*-ARHGEF3* or the control plasmid pcDNA3.1(+) using Lipofectamine 2000 (Invitrogen, Carlsbad, CA) according to the manufacturer's instructions. For the establishment of the *ARHGEF3*-HONE1 cell line stably expressing *ARHGEF3*, the cells were split at a ratio of 1:10 48 h after transfection. Next, cells were maintained in Leibovitz's L-15 medium containing 200 μg/mL of G418 (Calbiochem, San Diego, CA). After 6 weeks of selection, resistant colonies stably transfected with pcDNA-*ARHGEF3* (HONE1 pcDNA-*ARHGEF3*) or pcDNA3.1(+) [HONE1pcDNA3.1(+)] were pooled.

### *In vivo* tumorigenesis and metastasis assays

For the *in vivo* assays of subcutaneous tumorigenesis of NPC cells, 1×10^6^ mixed populations of HONE1-*ARHGEF3* cells stably overexpressing *ARHGEF3* or the control HONE1–vector were injected subcutaneously into the backs of 4-week-old male BALB/C-nu athymic nude mice. At day 30, the mice were sacrificed, the tumors were removed, and tumor weight was calculated.

To evaluate the metastasis potential of NPC cells *in vivo*, five 4-week-old male BALB/C-nu athymic nude mice in each experimental group were injected with HONE1-*ARHGEF3* and HONE1-vector cells, separately. Briefly, each mouse received 2×10^5^ cells via tail vein injection. Eight weeks after cell injection mice were killed and the lungs and the liver were removed from each mouse and fixed with phosphate-buffered neutral formalin. Consecutive tissue sections were made for each block of the tissues, which were then stained with haematoxylin-eosin. Finally, the slides of the lungs and the liver were carefully examined under a microscope. All experimental procedures involving animals were are accordant with the Guidelines for the Care and Use of Laboratory Animals (NIH publications Nos. 80-23, revised 1996).

### Apoptosis assay

To assess the rate of apoptosis, transfected cells were harvested and washed twice with cold PBS, and the Annexin V-PI Kit (Nanjing Keygen, Nanjing, China) was used according to the manufacturer's guidelines. The detection was performed with a FACS Calibur using Cell Quest software (BDIS, San Jose, CA).

### Real-time PCR gene array

RNA was extracted from si*ARHGEF3*-CNE2 and siNC-CNE2 cells using Trizol (Invitrogen, Carlsbad, CA) and was cleaned them using the RNeasy MinElute cleanup kit (Qiagen, Valencia, CA). Messenger RNA expression levels were quantified with a Human Tumor Apoptosis RT^2^ Profiler PCR array (Super Array Bioscience, Frederick, MD). Si*ARHGEF3*-CNE2 cells were compared with control siNC-CNE2 cells using a Human Apoptosis RT^2^ Profiler PCR array containing 84 key genes involved in programmed cell death.

### Statistical analysis

Statistical analysis was performed using the SPSS software package (SPSS Standard version18.0, SPSS Inc). Differences between variables were assessed by the Chi-square test or Fisher's exact test. For survival analysis, we analyzed all patients with NPC by Kaplan-Meier analysis. A log rank test was used to compare different survival curves. Multivariate survival analysis was performed on all parameters that were found to be significant in univariate analysis using the Cox regression model. Data derived from cell line experiments are presented as mean ±SD and assessed by the two-tailed Student's *t* test. *P* values of < 0.05 were considered statistically significant.
